# Isolation of *Enterococcus faecium* and determination of its mechanism for promoting the growth and development of *Drosophila*

**DOI:** 10.1038/s41598-023-43727-1

**Published:** 2023-10-31

**Authors:** Yujuan Li, Lei Pan, Pengcheng Li, Fuguo Gao, Lei Wang, Jian Chen, Zhichao Li, Yongheng Gao, Yumei Gong, Faguang Jin

**Affiliations:** 1 Department of Respiratory and Critical Care Medicine, Tangdu Hospital of Air Force Military Medical University, Xi’an, Shaanxi China; 2 Department of Surgery, Universitätsklinikum Erlangen, Friedrich-Alexander-Universität Erlangen-Nürnberg (FAU), 91054 Erlangen, Germany

**Keywords:** Developmental biology, Microbiology

## Abstract

Intestinal symbiotic microorganisms have a strong capacity to regulate the physiological functions of their host, and *Drosophila* serves as a useful model. *Enterococcus faecium* (*E. faecium*) is a member of the normal intestinal flora of animals. Lactic acid bacteria (LAB) such as *E. faecium* can promote the growth and development of *Drosophila*, but the mechanism of regulation of *Drosophila* is poorly understood. In this study, we found that *E. faecium* used a carbon source to produce probiotic acids. *E. faecium* is a symbiotic bacterium for *Drosophila*, and adult flies passed on parental flora to offspring. *E. faecium* promoted the growth and development of *Drosophila*, especially under poor nutritional conditions. *E. faecium* shortened the developmental process for *Drosophila* and accelerated the transformation from larva to pupa. Finally, *E. faecium* promoted the growth and development of *Drosophila* through TOR and insulin signalling pathways.

## Introduction

The human body, especially the intestinal tract, is inhabited by trillions of bacteria and other microorganisms^1,2^. The intestinal tract provides niches and nutrients for the microflora, which helps the host digest food, produce probiotics, and regulate a variety of host physiological activities^3^. Indeed, the host and the microflora establish a mutually beneficial symbiotic relationship that is essential for human health, ecology, and genetic variation^4^, but the complexity and diversity of the flora hinders in-depth analysis of its mechanisms^5,6^.

In recent years, *Drosophila*, has attracted increasing attention as a model for studying symbiotic relationships between hosts and bacteria due to the ease of establishing aseptic and intact systems, and its high degree of conservation with human flora and intestinal structure^7^. Like a mammal, the intestines of conventionally reared (CR) *Drosophila* are also home to numerous symbiotic microorganisms, making it a good model for asepsis (germ free, GF) or gnotobiotic analysis^8^. Recent studies have shown that intestinal microorganisms have important effects on the growth and development of *Drosophila*^7,9^*.* Firstly, *Drosophila* eat rotten fruit, which contains a large number of fermentative microorganisms^7^, indicating that these microorganisms may be beneficial to the development and growth of *Drosophila*^10^. Secondly, *Drosophila* ovulates in vitro, and the egg is covered with a layer of chitin, which can resist certain physical and chemical damage, making it easy to establish a sterile host via body surface disinfection. Based on a GF approach, a gnotobiotic model was established by inoculating specific bacteria^7^. Thirdly, the intestinal microbial structure of *Drosophila* is similar to that of human intestinal microorganisms^11^. Finally, our in-depth knowledge of genetics in *Drosophila* makes it convenient for us to analyse the molecular mechanism of microbial regulation of the host.

*Enterococcus faecium* is a Gram-positive bacterium and a member of the normal intestinal flora of animals. Because it can produce lactic acid, it belongs to the lactic acid bacteria (LAB). Studies have confirmed that there are more than 20 kinds of bacteria in *Drosophila* caught in the wild and raised in the laboratory, and LAB are among the most common classes of symbiotic bacteria in *Drosophila*^12^. Food spoilage caused by *E. faecium* facilitates host growth and reproduction, favouring the survival of semi-saprophytic insects such as fruit flies in nature, creating a new niche for them and increasing species diversity. However, a species of symbiotic *E. faecium* and the mechanism by which they promote the growth and development of *Drosophila* remain largely unknown. In this study, *E. faecium* was isolated from the intestine of *Drosophila* using selective medium, and the mechanism for promoting *Drosophila* growth and development was investigated.

## Results

### Isolation and identification of bacteria from the intestinal tract of *Drosophila*

**Figure 1:**
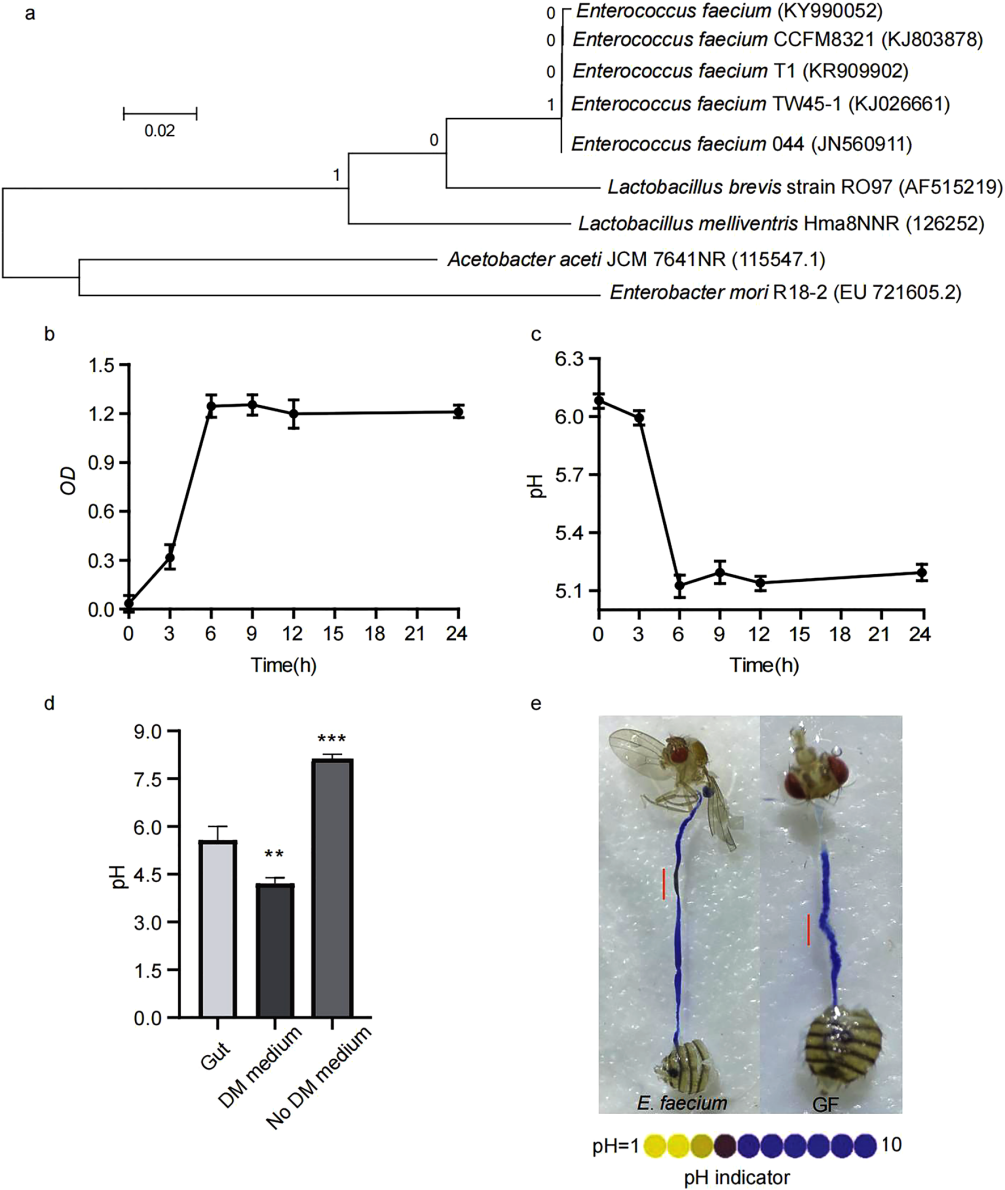
Phylogenetic tree of *Enterococcus faecium* and relatives and its in vitro features of culture. (**a**) Phylogenetic tree of *Enterococcus faecium* and its homologies. Bar: nucleotide divergence; number at notes present bootstrap percentages; those in parentheses are GenBank accession No. (**b**) Growth of *Enterococcus faecium* with carbon sources in medium. (**c**) pH curves of *Enterococcus faecium* growing in medium with carbon sources. Values represent mean ± SEM. (**d**) Determination of pH in the gut of CR *Drosophila Melanogaster* (n = 2000), with (DM medium) and without (no DM medium). (**e**) Expression of acidic results in CCR. Acidic regions indicated by pH indicator Bromophenol blue.

The strain of bacteria was isolated from the intestines of *Drosophila* using selective medium^13^. The bacterium was confirmed to be Gram-positive and facultative anaerobic, and its morphological and biochemical characteristics were consistent with those of *Enterococcus*. Sequencing results showed that the 16S rRNA gene was 1103 bp, and the isolated strain was confirmed to be an *E. faecium* strain (GenBank accession number: KY990052) based on its closest genetic relationship with *E. faecium*. MEGA 6.0 software (http://www.downxia.com) was used to analyse the homology of genes and construct a phylogenetic tree (Fig. 1a). The sequences of *E. faecium* KY990052, *Enterococcus* isolated from human faeces CCFM8321 (KJ803878) and *E. faecium* T1 (KR909902) in cheese shared the highest homology (99%), indicating that the three strains had the closest genetic relationships. However, there was no significant similarity in the gene sequences of *Acetobacter aceti* and *Enterobacter mori* commensal to the gut of *Drosophila*, indicating a distant genetic relationship.

### In vitro culture characteristics of *E. faecium*

*E. faecium* KY990052 cells grew rapidly in YCFA liquid medium with 0.25% glucose, with a logarithmic growth phase from 3 to 9 h (Fig. 1b). At 9 h, the *OD*_*600*_ value was 1.1 ± 0.3 near the peak, and it decreased slightly thereafter. After 3 h of culture, the pH value of the culture medium began to decrease continuously (Fig. 1c), and the lowest value was 5.1 ± 0.1. *E. faecium* KY990052 is a typical lactic acid-producing coccus, which can also reduce the pH value in the intestine and maintain its acidic environment. As shown in Fig. 1d, the pH of the gut of CR fly is 5.5, the pH of the single medium is 8.1, and the pH of the medium containing fly is 4.2. The latter two are statistically significant compared with the pH of the gut. These results indicated that CR *Drosophila* could significantly reduce the pH value of the medium. The midgut of flies contains an acidic gastric or copper cell region (CCR,^14^), which controls distribution and composition of the microbiota, and control the survival of ingested bacteria^15^. We detected that the pH of *E. faecium* associated intestinal CCR region was 4, and that of GF group was 7 (Fig. 1e), which was similar to that of in vitro fermentation experiment.

### *E. faecium* colonises the *Drosophila* gut

**Figure 2:**
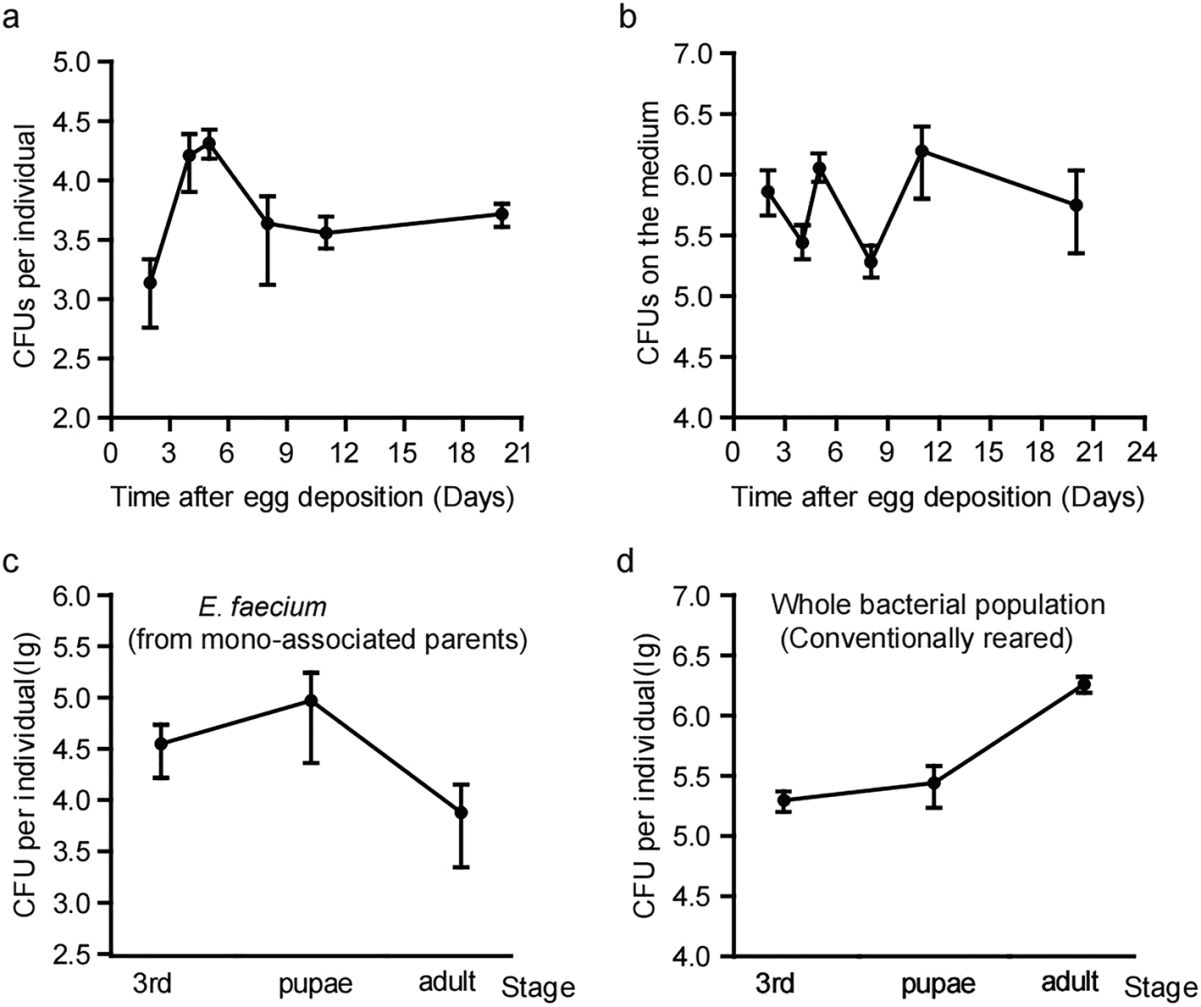
*E. faecium* was commensal bacteria of *Drosophila*. (**a**) The mount of internal bacterial loaded of *E. faecium* in various growth stages of the gut of *Drosophila*. (**b**) The mount of internal bacterial loaded of *E. faecium* in various growth stages of the culture medium of *Drosophila*. (**c**,**d**) The mount of internal bacterial loaded of the progenies of *Drosophila* adults mono-associated with *E. faecium* and CR*.* Values represent mean ± SEM.

In order to determine which symbiotic bacteria mediate this effect, we analysed the characteristics of bacterial communities associated with CR fly strains. As shown in Fig. 2a, the bacterial load in the intestines of 3rd instar larvae, pupae and adults was ≥ 10^3^ and more on the 1st, 4th, 12th and 27th day after eclosion, indicating that *E. faecium* could effectively colonise the intestines of *Drosophila*. The bacterial density of the culture medium was higher, and the bacterial density per gram of medium was ≥ 10^5^ in the larval and pupal stages, and the corresponding period at 1, 4, 12 and 27 days after adult eclosion (Fig. 2b), indicating that *E. faecium* could exist stably in the culture medium.

Since vertical transfer is typical for microbiota acquisition, we tested whether *E. faecium* could be successfully transmitted from parents to offspring^12^. Figure 2c shows that *E. faecium* could be stably colonised in the 3rd instar larval, pupal and adult stages of the G1 generation of fruit flies, and the bacteria-carrying capacity in the intestines of these three stages were 10^4^, 10^5^ and 10^3^, respectively, indicating that *E. faecium* could be vertically transmitted from generation to generation. The above results show that *E. faecium* is closely associated with *Drosophila*, and can be considered symbiotic. Similar dynamics for sustentation of the whole bacterial population and colony-forming units (CFUs) were observed during the larval, pupal and early adult stages of CR individuals (Fig. 2a,d). These results indicate that the protocol used to associate GF individuals with *E. faecium* accurately revealed the natural pattern of bacterial colonisation of CR during larval, pupal and early adult stages. Thus, *E. faecium* has a strong ability to colonise the whole larval niche of the host intestine, and maintain a population in the host digestive tract.

### *E. faecium* association sustains larval development under nutrient scarcity

**Figure 3:**
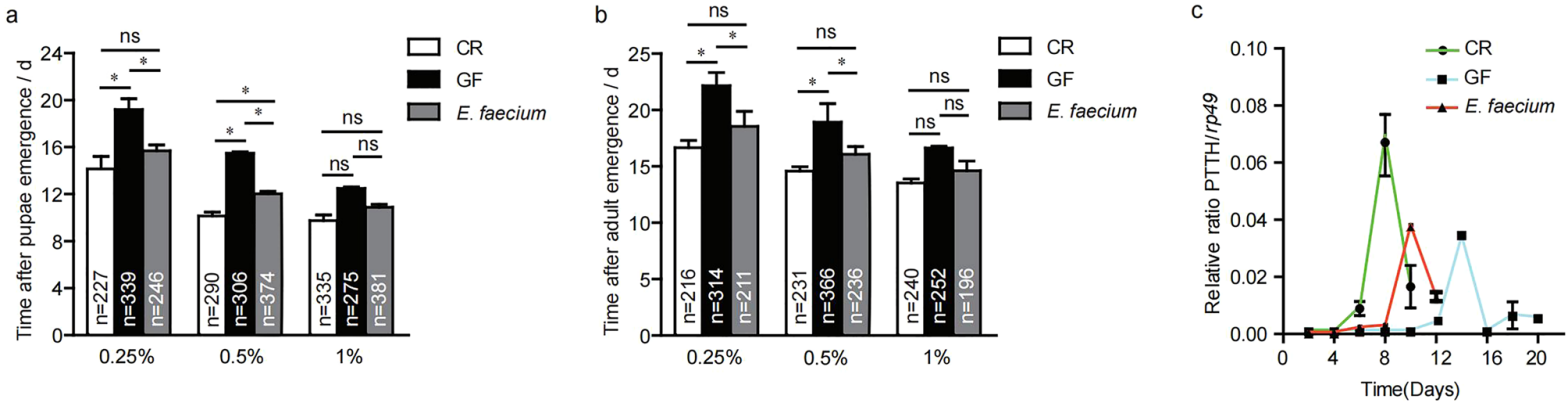
The *E. faecium* promoted the growth and development of *Drosophila*. (**a**) The timing of pupa formation of CR, GF or *E. faecium* in the different concentrations of yeast. (**b**) The timing of the emergence of adult of CR, GF or *E. faecium* in the different concentrations of yeast. (**c**) *PTTH* mRNA levels for CR, GF and *E. faecium-*associated *Drosophila.* (CR VS GF, *P* < 0.05, CR VS *E. faecium*, *p* > 0.05, GF VS *E. faecium*, *p* < 0.05). ns *P* > 0.05, *P < 0.05; values represent mean ± SEM.

Having proved that *E. faecium* colonised the larval niche as a symbiotic bacterium, we tested whether *E. faecium* could promote the development of larvae raised on low-nutrient medium. The combination of *E. faecium* and nutrient-poor medium was enough to accelerate the growth of larvae, and both pupae and the adults fly eclosed earlier (Fig. 3a,b). However, this effect was not observed in medium rich in nutrients. This shows that *E. faecium* has a specific effect on the growth of whole body larvae, reflecting not only the nutritional effect of adding microorganisms to the fly culture medium, but also the specific biological activities of these strains. These observations showed that *E. faecium* accelerated the development of larvae, and led to the emergence of adults earlier under nutrient-poor conditions.

In *Drosophila*, prothoracicotropic hormone (*PTTH*) secreted by the brain initiates the secretion of the steroid hormone hydroxyecdysone (20HE), which promotes the transition from 3rd instar larva to pupal stages^16^. Expression of the *PTTH* gene is regulated over time, and expression is higher in the late third instar and early pupal stages, hence expression serves as a molecular index to evaluate the development of *Drosophila*. The transcriptional level of *PTTH* in the brain of *Drosophila* was measured by the RT-PCR technique. Figure 3c shows that expression of *PTTH* peaked on the 8th day for CR, but for GF it did not reach its peak until 14 days; GF was ~ 6 days slower than CR, and expression for CR was higher than that for GF (*p* < 0.001). Expression of *PTTH* reached its peak on the 5th day for *E. faecium*, 4 days earlier than for GF, and levels were higher than or GF (*p* < 0.05). The peak was close to that for CR, which indicates that *E. faecium* effectively rescued the growth retardation of GF, and accelerated the transition from larval to pupal stages.

### *E. faecium* promotes larval growth

**Figure 4:**
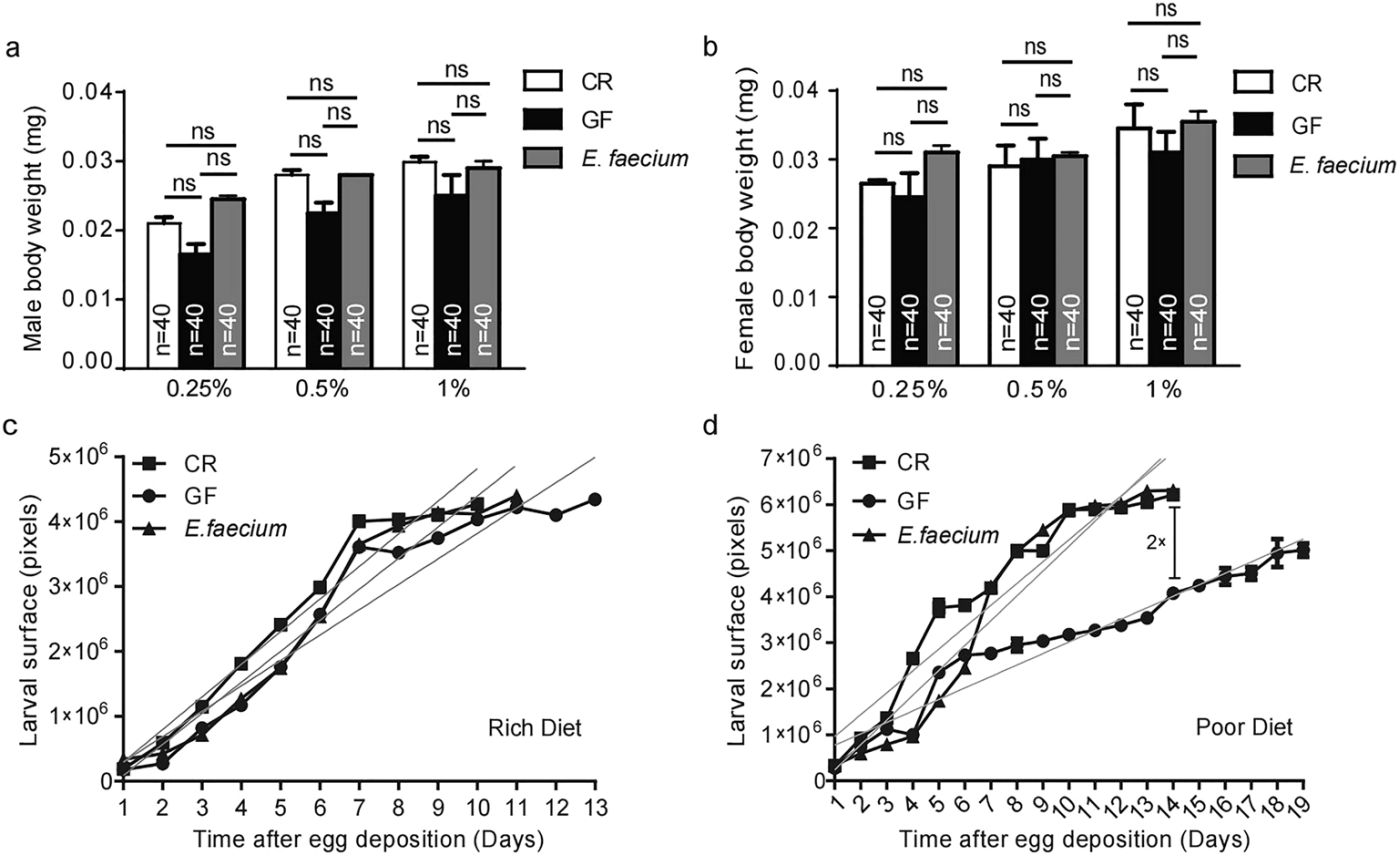
The *E. faecium* improved the growth ratio of larval, but it had little effects on weight. (**a**,**b**) The weight of male and female *Drosophila* on different concentrations of medium. (**c**,**d**) Larval surface of CR or GF, or *E. faecium* associated larvae over time when grown on rich (1.0% yeast) or poor diet (0.5% yeast). Linear regression curves are included. CR/Poor Diet, Y = 693,900X – 219,500; GF/Poor Diet, Y = 410,400X – 349,700; *E. faecium*/Poor Diet, Y = 740,600X – 260,100; CR/Rich Diet, Y = 855,100X − 182,500; GF/Rich Diet, Y = 516,000X − 156,100; *E. faecium*/Rich Diet, Y = 806,500X – 627,300. Values represent mean ± SEM (ns *p* > 0.05; *p < 0.05). A Under the condition of poor medium, the pupation time of CR and *E. faecium*-associated larvae was twofold short than GF.

In order to further assess the effects of *E. faecium* on the growth of larvae, we analysed the size of adults, which is the final parameter for the growth stage of larvae. To this end, we compared the body weight of adults raised on medium containing CR, GF or *E. faecium*. For the duration of the larval stage, we did not observe a difference in the weight of adults growing on nutrient-rich medium. Similarly, when larvae grew on nutrient-poor medium, no significant differences were observed between CR, GF and *E. faecium-*associated individuals (Fig. 4a,b). However, adults developed from GF, GF and *E. faecium*-associated larvae grown under poor dietary conditions weighed less than adults grown under rich conditions (Fig. 4a,b).

Because *E. faecium* shortens its growth stage without impacting the size of adults, we assume that *E. faecium* increases the growth rate of larvae. To confirm this, we compared the size of individuals associated with CR, GF and *E. faecium* from L1 larval to pupal stages. There was no difference in pupation rate of CR, GF and *E. faecium*-associated larvae fed a rich diet (Fig. 4c), but the pupation rate of CR-related larvae fed a poor diet was two-fold higher than that of the GF group, and the results for *E. faecium*-associated larvae were similar to those of the CR group (Fig. 4d), confirming the results for Fig. 3c. These findings demonstrated that under conditions of nutrient deficiency, *E. faecium* promoted individual growth by increasing the pupation rate of larvae and shortening the growth cycle, which significantly shortened the pupation time of GF larvae.

### Effect of *E. faecium* on intestinal cell proliferation

**Figure 5:**
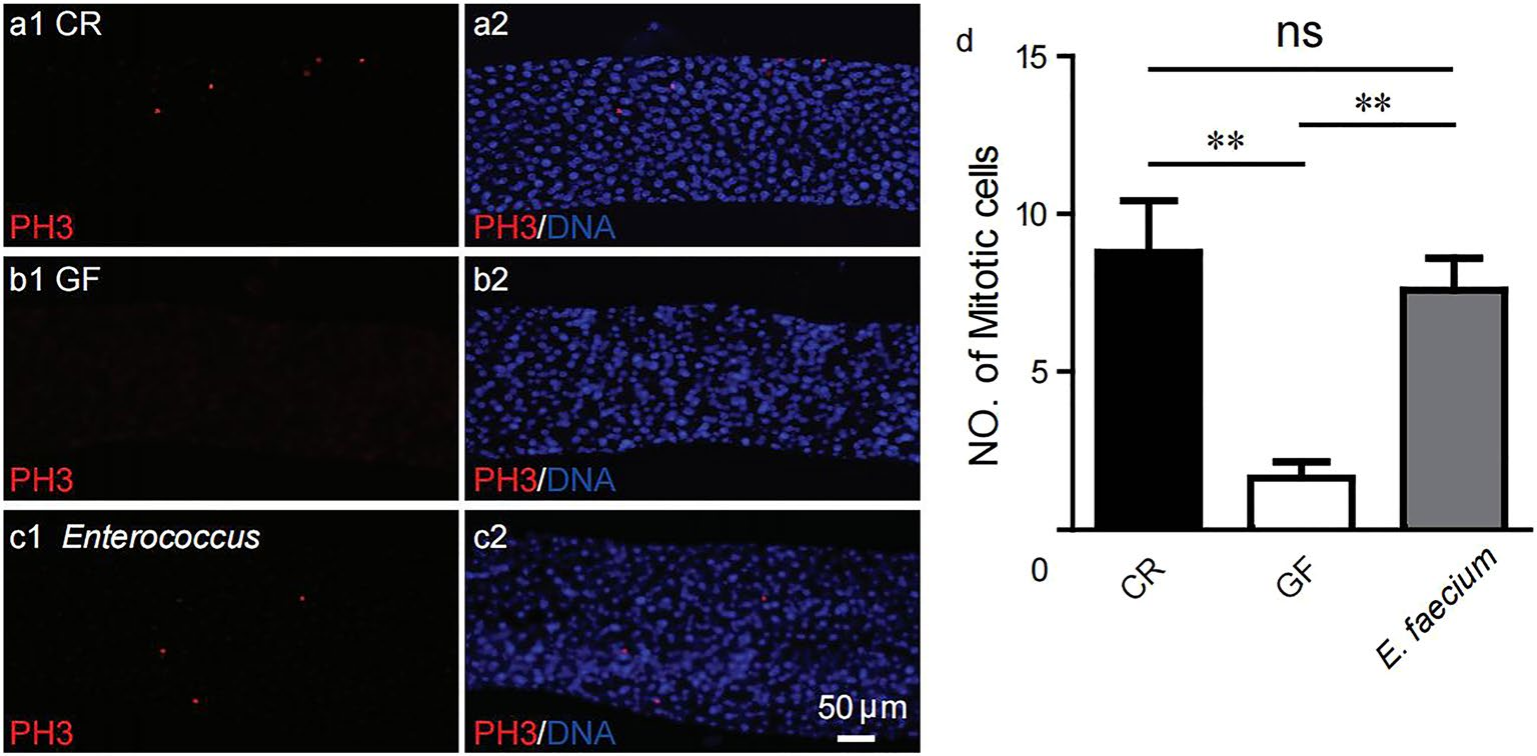
*E. faecium* promoted the proliferation of *Drosophila* intestinal cells. (**a**–**c**) The representative imaging of mitotic cells in guts of CR, GF and *E. faecium* flies (red as PH3 marker, blue as DAPI marker, bar 50 μm). (**d**) The quantification of mitosis in gut region of CR, GF and *E. faecium* flies. The anterior gut, midgut and posterior are parts of gut regions, n = 10. Values represent mean ± SEM. ns *p* > 0.05, **p < 0.01.

It has been reported that the main symbiotic bacteria in the intestinal tract of *Drosophila* can control the development rate, size, energy metabolism and intestinal stem cell activity of *Drosophila*^17^. Adult stem cells play a crucial role in organ homeostasis and palingenesis through their ability to maintain self-renewal and generate differentiated cells. The adult midgut of *Drosophila* is a frequently-used model system for stem cell biology^18–21^. This tissue undergoes rapid cell renewal by intestinal stem cells (ISCs), the sole mitotic cells in the gut. They generate diploid enteroblasts (EBs), which differentiate to polyploid enterocytes or diploid EEs^22^. We used anti-phosphorylated histone 3 (PH3) antibodies to evaluate the number of mitotic cells to determine mitotic activity^23^. The results in Fig. 5 show eight cells in the metaphase of midgut division for CR, compared with two cells for GF (*p* < 0.001), and the number of *E. faecium* cells was significantly higher than for GF (*p* < 0.001), which significantly rescued the defective intestinal cell division and proliferation of GF.

### Effects of *E. faecium* on the steroid hormone ecdysone and regulation of the insulin signal pathway

**Figure 6:**
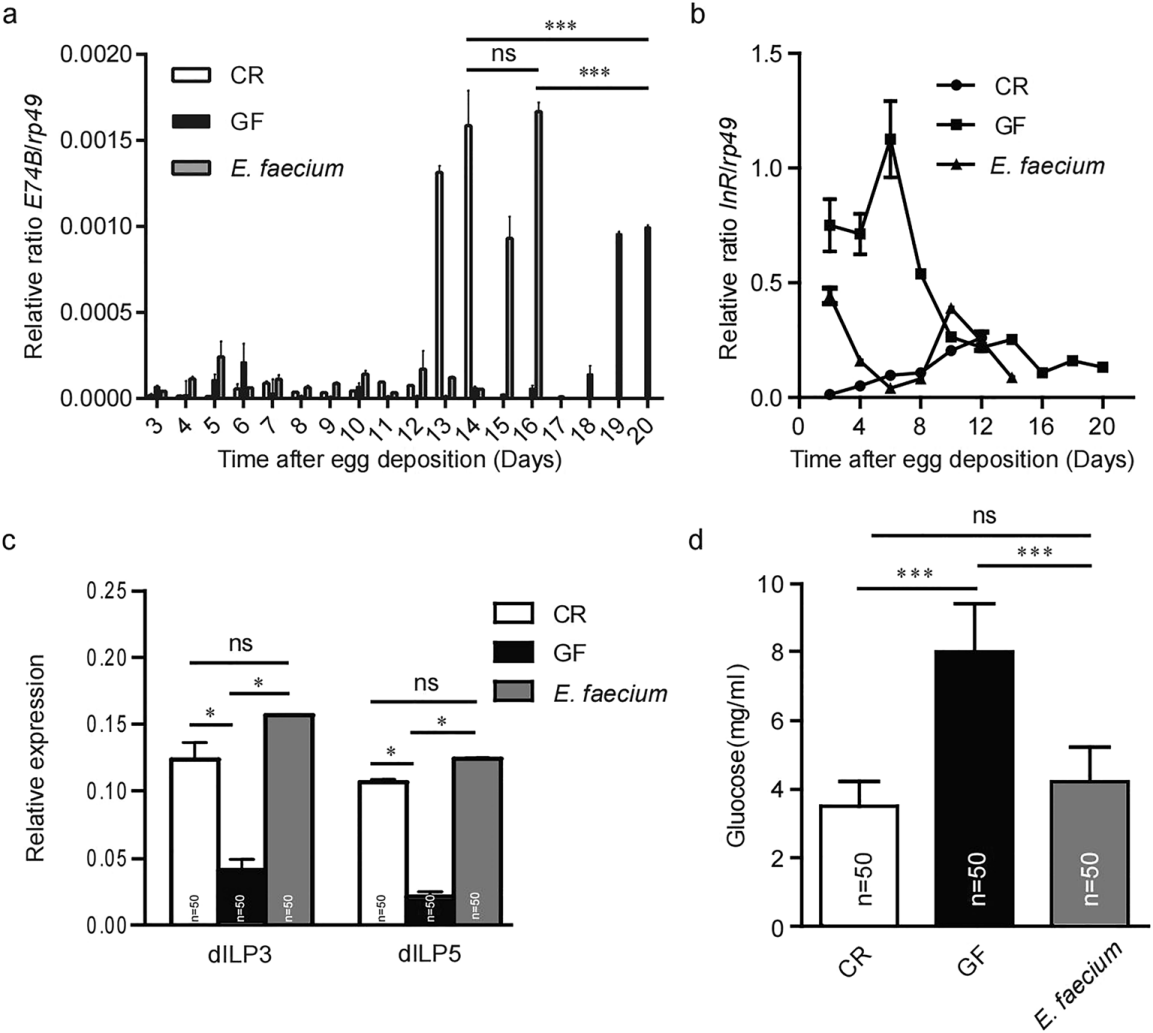
The *E. faecium* promoted the secretion of hormone and was required to regulate the insulin signal pathway. (**a**–**c**) *E74B*, *InR, dILP3* and *dILP5* mRNA levels for CR, GF and *E. faecium-*associated *Drosophila.* (**d**) Glucose levels for CR, GF and *E. faecium-*associated *Drosophila.* Values represent mean ± SEM. ns *p* > 0.05; *p < 0.05, ***p < 0.001.

In *Drosophila*, the larval period and the larval growth rate are controlled by two crucial hormones: the steroid hormone ecdysone (Ecd) and *Drosophila* insulin-like peptides (dILPs), respectively^24^. Transcription factor *E74B* is one of the early genes to act in response to increased Ecd concentrations, and it serves as a molecular marker of Ecd activity^25^. To determine whether *E. faecium* directly affects these growth signals, we compared the strength of these signals in CR, GF and *E. faecium*-associated larvae. Figure 6a shows that for CR, the highest mRNA levels for *E74B* were reached at day 14 after oviposition, while for GF, peak expression of this gene was significantly delayed until day 20 after oviposition, and the difference was statistically significant (*p* < 0.001). Transcriptional levels peaked on day 16 in *E. faecium-*associated larvae, hence the delayed expression of the gene in GF was clearly rescued. However, levels of *E74B* mRNA peaked sharply in CR and *E. faecium-*associated larvae, while GF larvae showed a lower peak that was delayed. Interestingly, in GF larvae, levels of *E74B* mRNA were already increased on day 6 (although this was not statistically significant), and there was no pupariation at this time (pupariations first observed on day 23; Fig. 3b). These results showed that *E. faecium-*associated 3rd instar larvae showed early and stronger Ecd peaks, which rescued the delayed Ecd expression of GF.

We next measured *InR* gene expression levels as a marker for intracorporal dILP activity. *InR* gene transcription reflects negative regulation of the *InR* signalling pathway through the activity of the fork head transcription factor (dFOXO). The *InR* gene is a negative regulator of intracorporal dILP activity; lower *InR* expression is associated with greater dILP activity^26^. As shown in Fig. 6b, *InR* expression was consistently lower in CR larvae than in GF larvae. The *E. faecium*-associated larvae were effectively rescued by over-expression of the *InR* gene in GF, suggesting that *E. faecium* bacteria are equivalent to CR associated with increased intracorporal *InR* signalling during the period of larval growth. We then measured *dILP3* and *dILP5* gene expression as a positive marker for insulin signal pathway activity. As shown in Fig. 6c, *dILP3* and *dILP5* expression levels were higher in CR larvae than in GF larvae. *E. faecium*-associated larvae were effectively rescued from low expression of *dILP3* and *dILP5* genes in GF. These results showed that *E. faecium* is associated with increased dILP activity during larval growth, and this is similar to the effect of CR.

It is known that insulin/insulin-like growth factor signalling (IIS) mutant animals display diabetic phenotypes, with increased levels of circulating sugars^27–31^. As shown in Fig. 6b,c, GF showed abnormal expression of *dILP3*, *dILP5* and *InR* genes. In the Fig. 6d, we also measured levels of sugars in CR, GF and *E. faecium-*associated larvae, and detected elevated levels of total sugars (glucose) in GF compared with CR larvae (*p* < 0.001), and glucose was the major disaccharide in insects. *E. faecium* effectively reduced the higher levels of glucose in GF, and there was no difference compared with CR. Taken together, our results support the notion that although differing in terms of kinetics, *E. faecium-*associated larvae strengthen the systemic generation of two hormonal growth signals.

## Discussion

Due to the relative simplicity of the microbial community and facile establishment of the bacterial host, *Drosophila* provides a useful model that avoids complexity and is suitable for revealing the molecular mechanisms by which microflora modulate host physiology. Our current results showed that intestinal microorganisms had an important effect on the development of *Drosophila*. The bacterial community in the digestive tract of *Drosophila* is relatively simple; the dominant bacteria are *Firmicutes* (*Acetobacter* and *Lactobacillus*). Given the great differences in the growth environments of fruit flies, it is possible that their symbiotic microbes are much more complex than expected. Herein, *E. faecium* KY990052 was isolated from the intestinal tract of *Drosophila* for the first time, revealing greater diversity and complexity of the intestinal bacteria of *Drosophila* (Fig. 1). This strain can effectively colonise the intestinal tract of *Drosophila* and be passed from parents to offspring, suggesting that *E. faecium* is a symbiotic organism of *Drosophila* (Fig. 2). At the organism level, *E. faecium* increased the growth rate of *Drosophila*, promoted *Drosophila* growth, and accelerated the eclosion rates of pupae and adults (Fig. 3). At the molecular level, *E. faecium* enhanced the production of two key growth hormones (Ecd and dILPs) in the larval stage, thus promoting the growth rate of the larval stage, and ultimately the growth and development of *Drosophila* (Fig. 6).

LAB are Gram-positive bacteria commonly found in the gastrointestinal tracts of animals, fermented food, dairy products, and natural environments including soil and water^32^. LAB are also common symbiotic microorganisms of *Drosophila*, including *Lactobacillus* and *Enterococcus*. *Enterococcus* is a large genus of LAB. In *Drosophila*, many species have been identified as commensal bacteria, including *E. faecium*^33^. LAB convert carbohydrates from raw materials into lactic acid, and they are often used in food fermentation to obtain a variety of high-quality end products^34^. Studies have shown that *Enterococcus* participates in and acts on a variety of physiological processes in the host^12,35–37^, but there are few reports on how it affects the development of the host. *E. faecium* decomposes nutrients in fruit using its own enzyme system, and the decay caused by *E. faecium* is not only beneficial to its own reproduction, but also provides more available nutrients for fruit flies, and creates a new niche for the host^37^. This is beneficial for the survival and reproduction of fruit flies. *Drosophila* are semi-saprophytic insects that mainly depend on eating rotten fruit, and they form a mutually beneficial symbiotic relationship with *E. faecium* in the natural environment.

This symbiotic relationship is reflected in the fact that *E. faecium* promoted the growth and development of *Drosophila*. Ours and other studies showed that *Drosophila* cannot survive without microorganisms, even when given adequate nutrients such as protein and glucose, indicating that microorganisms are necessary for the growth and development of *Drosophila*^5^. Members of the *Drosophila* intestinal flora can affect the genetic factors of some animals and the composition of important nutrients in the body, such as triglyceride (fat) content; high diversity among intestinal microorganisms can improve the digestion and absorption of nutrients and increase nutritional reserves in the host body^38^. Many animals have an intestinal region with a low pH to aid protein digestion, absorb nutrients such as calcium, iron and vitamin B_12_, and kill enteric pathogens and parasites acquired orally^39^. Our current study showed that *E. faecium* can utilise carbohydrates (glucose) and lower the pH to 5.1 (Fig. 1). Furthermore, *E. faecium* is resistant to bile salts and poor gastrointestinal conditions. Its main function is automatic aggregation and adhesion, and it synthesises a variety of bacteriocins called enteroglobulins; these small, low-molecular-weight, heat-resistant, ribosomal antibacterial peptides possess antibacterial activity^40,41^. Microorganisms in the intestinal tract of *Drosophila* promote immune activity by stimulating innate immune signals of intestinal epithelial cells^42^. Herein, we explored the signalling mechanism by which *E. faecium* promotes the growth and development of *Drosophila*.

The insulin signalling pathway dynamically regulates the transcription and translation of many proteins involved in glucose uptake, promotes intestinal cell division and proliferation, and regulates the whole process of body development, growth and metabolism^7^. It has been reported that the main symbiotic bacteria in the intestinal tract of *Drosophila* can regulate insulin signalling and the target of rapamycin (TOR) signal pathway, the host self-balance program, and control development rate, size, energy metabolism and intestinal stem cell activity^9,12,17,43^. In *Drosophila*, the TOR pathway regulates the hormone signal of larval growth in a tissue-specific manner, and the production of Ecd is controlled by the prothoracic gland in the middle stage of the 3rd instar larvae^42^. *E. faecium-*associated larvae can also express the *E74B* gene in advance, which rescues the expression defect of GF (Fig. 6a). Systemic *InR* signalling is regulated by remote control of dILP secretion by neurons, which is regulated by TOR activity in the fat body^44,45^. We found that the LAB *E. faecium* is a major symbiotic organism in the intestinal tract of *Drosophila.* Figure 6b,c show that *E. faecium* rescued defective expression of insulin signalling pathway genes (*InR*, *dILP3* and *dILP5*) in GF, which controlled levels of sugars in the body (Fig. 6d), and had a similar effect to CR. The results showed that *E. faecium* could promote the development of *Drosophila* through the insulin signal pathway alone. This TOR signalling activity in the fat body triggered whole body *InR* signal transduction and increased the growth rate. In the prothoracic gland, TOR signalling enhanced the production of Ecd in the latter stages of larval development, and shortened the length of the growth stage. The combined effects of increased TOR activity and hormone signals led to optimal whole body growth. We also found that *E. faecium* increased the number of mitotic cells in the midgut of *Drosophila* (Fig. 5), which helped to maintain homeostasis and functioning in the host intestine, and created the necessary conditions for the absorption and utilisation of nutrients.

The above results showed that *E. faecium* promoted the growth and development of *Drosophila*, and increased the species diversity of intestinal microorganisms. This diverse intestinal microflora participates in the digestion, absorption and synthesis of nutrients. *Drosophila* provides a good model for intestinal microorganisms in humans and other animals. Using *Drosophila* as a model to study the role of symbiotic bacteria can provide important information pertinent to human diseases.

## Materials and methods

### Strain culture and *Drosophila melanogaster* breeding

All *Drosophila* were reared at 25 °C in 50–60% humidity under a 12 h/12 h light/dark cycle. The Oregon R strain was used for wild-type flies. *Drosophila* were raised on standard cornmeal sugar agar medium (Agar 7.5 g, grape juice 58 ml, sucrose 8.2 g, yeast 58.8 g, corn meal 58.8 g, distilled water 1000 ml)^46^.

### Isolation and identification of bacteria

Fifteen CR-associated adults were anesthetised with carbon dioxide, placed in tubes, disinfected twice with 75% ethanol, washed twice with phosphate-buffered saline (PBS), ground, and cultured in a 100 μL volume at 37 °C for 24 h on plates. Single colonies were selected and cultured in liquid medium for 24 h. DNA was extracted by pyrolysis and PCR was carried out on the 16S rRNA gene using universal primers. The 16SrRNA gene sequence was submitted to GenBank to obtain the registration number of the strain. The 16S rRNA gene sequences of nine strains were downloaded from the GenBank database. Based on the homology of the 16SrRNA gene, MEGA 6.0 software was employed to generate a phylogenetic tree using the neighbour-joining method.

### Bacteria culture and cell counting

Bacteria were isolated from *Drosophila* using specific media from the China General Microbiological Culture Collection Center, and identified from 16 SrRNA sequences with a PCR primer set^47^. To culture commensal bacteria, selective media were used to assay the bacterial population of *E. faecium* in 200 ml of liquid YCFA medium with 0.25% glucose^48^.

### In vitro culture of *E. faecium*

Isolated *E. faecium* cells (2.5%) were injected into YCFA liquid medium containing 0.25% glucose (1L medium contains 10 g tryptone, 2.5 g yeast extract, 4 g NaHCO_3_, 1 g cysteine, 1 g inulin, 0.45 g K_2_HPO_4_, 0.9 g NaCl, 0.09 g MgSO_4_·7H_2_O, 0.09 g CaCl_2_, 1 mg resazurin, 10 mg haemin, 10 μg biotin, 10 μg cobalamin, 30 μg p-aminobenzoic acid, 50 μg folic acid, 150 μg pyridoxamine, 50 μg thiamine, 50 μg vibioflain, 13.8 mM glucose) with H_2_/CO_2_ in the gas phase^47^, and its cultured in a 37 °C incubator, and sampled at 0, 3, 6, 9,12 and 24 h for *OD*_*600*_ and pH measurement.

### pH indicator, blue dye

Putting 150ul of 2% Bromophenol blue sodium (pH indicator, Sigma) was added to a food vial, and stir the blue fuel with a sterile rod. Flies were fed for 10 h. For *E. faecium* feeding, flies were fed 200 ul of 1 *OD* bacteria (in 5% sucrose) mixed with 100ul 2% pH indicator in filter paper for 24 h.

### Establishment of germ-free (GF) and gnotobiotic populations

The GF model has been described previously in the literature^9,49^. Based on GF, 1 *OD* of *E. faecium* (take 100 μl of the bacterial solution, about 10^5^ density) solution was added to medium and a gnotobiotic model of *Drosophila* (100 μl embryo) was established^49^. At least five tubules were included in each group.

### Colonisation and generation transmission of *E. faecium *in *Drosophila*

Larvae at 2nd to 3rd instar stages were taken from 0 to 6 days after oviposition, along with pupae from 6 to 8 days and adults emerging at 1, 4 and 12 days. *Drosophila* were disinfected twice with 75% ethanol, washed twice with PBS, and ground. A step dilute release coating plate (YCFA) was placed in a 37 °C incubator for 48 h to observe and count colony growth (CFU/gut). In order to avoid the effects of yeast meal containing microbial factors on *Drosophila* development, casein hydrolysate was used to replace yeast meal^9^. At the corresponding growth stage of *Drosophila*, 1 g casein culture medium was added to the coating plate, which was diluted and cultured at 37 °C for 24 h. Colony growth was observed and counted (CFU/g).

CR or *E. faecium*-associated adults were transferred to casein medium after strict bacteria-free handling, and discarded after oviposition. After pupation from eggs, they were disinfected with 75% ethanol twice, cleaned with phosphate buffer twice, and transferred to sterile 4% yeast medium. After emergence and oviposition, the adult G0 generation was discarded. The intestinal bacteria content of 3rd larvae, pupae and adults of G1 generation was detected, using the same detection method as the colonization experiment (CFU/gut).

### Determination of developmental duration and growth rate of *Drosophila*

The number of CR, GF and *E. faecium-*associated pupae and adults in 0.25%, 0.5% and 1% (casein%) casein medium was recorded, and the time of pupae formation and adult emergence was determined. There were at least 20 flies per tubule. The following equation was used:

$${\text{T }} = \, \left( {{\text{T1 }} \times {\text{ N1 }} + {\text{ T2 }} \times {\text{ N2 }} + \cdots + {\text{ Tm }} \times {\text{ Nm}}} \right)/({\text{N1 }} + {\text{ N2 }} + \cdots + {\text{ Nm}})$$

where T is the development period of the corresponding stage, Tm is the days from spawning to the corresponding stage, and Nm is the number of *Drosophila* in the corresponding stage on day Tm. Casein medium at three concentrations comprised 0.5 g/L sucrose, 0.7 g/L corn flour, 0.1 g/L agar, and 0.025 g/L, 0.05 g/L or 0.1 g/L casein.

On the 3rd day after adult emergence, 40 fruit flies in the three groups (CR, GF and *E. faecium*) were weighed. Ten fruit flies (0 days larvae to pupae) were placed on a glass slide, frozen at − 20 °C for 1 h, images were taken under a DM4000 microscope (Leica, Wetzlar, Germany), and ImageJ software (https://imagej.en.softonic.com/) was used to measure the area.

### Immunofluorescence staining

Ten CR, GF and *E. faecium*-associated adults (emergence 3 days) were selected, their intestines were dissected in PBS, and fixed in 4% paraformaldehyde solution at room temperature for 30 min. 0.3% PBST (Triton X-100) containing 0.2% goat serum and 0.1% fetal bovine serum was added and incubated for 30 min, rabbit primary antibody PH3 (Millipore, H0412; 1:1000) was added and incubated overnight at 4 °C, and anti-rabbit secondary antibody (Invitrogen, WP20007, 1:1000) was added and incubated for 2 h. PBST was used to wash samples. DAPI (Invitrogen, 1:1000) staining for 10 min, PBST washing, tablet sealing, microscopy image collection (Leica DM4000), and statistical analysis were then performed. Finally, the average value was taken.

### Real-time PCR

Ten CR, GF and *E. faecium-*associated *Drosophila* were selected from 0.5% casein medium (3-day larvae to 3-day pupa) were selected and total RNA was extracted by the TRIzol method. A 5 μg sample of RNA was used as template, and oligo-DT primer was used for reverse transcription of mRNA to synthesise cDNA using a Prime Script RT reagent Kit (TaKaRa). Primer sequences are provided in previous work^16,25,26^. Primer sequence: *PTTH* (F:AACAGTGGCGGATTCGGTT, R:TACTCGGAGCATTGGAGGCAT), *E74B* ( F: GAATCCGTAGCCTCCGACTGT, R:AGGAGGGAGAGTGGTGGTGTT), *InR* (F: AACAGTGGCGGATTCGGTT, R: TACTCGGAGCATTGGAGGCAT), *rp*49 (F: GACGCTTCAAGGGACAGTATCTG, R: AAACGCGGTTCAGCATGA), *dILP3* (F: AAGCTCTGTGTGTATGGCTT, R: AGCACAATATCTCAGCACCT), *dILP5* (F: AGT3TCTCCTGTTCCTGATCC, R: CAGTGAGTTCATGTGGTGAG). Each group did 10 technical replicates and 3 biological replicates. Results were calculated using the formula ΔCt = Ct (target gene) − Ct (reference gene), and relative expression levels were determined using the 2^–ΔΔ*Ct*^ method.

### Sugar measurement

Fifty CR, GF and *E. faecium*- associated 3rd instar larvae were collected, rinsed three times in sterile water, ground, centrifuged at 4000*g* for 2 min, and 2 μL of the supernatant was treated with a glucose kit (Sigma, St. Louis, MO, USA) to determine the absorbance value (wavelength 505 nm).

### Data analysis

The experiment was repeated three times or more, and the average value for each group was calculated and subjected to Student’s *t-*test. Results are presented as mean ± standard error of the mean (S.E.M.). *p < 0.05, **p < 0.01, ***p < 0.001.

## Data Availability

The datasets generated during the current study are available in the [Genbank] repository, The Genbank accession number: KY990052 and the Website: [https://www.ncbi.nlm.nih.gov/nuccore/KY990052].
